# On the Stability of Lung Parenchymal Lesions with Applications to Early Pneumothorax Diagnosis

**DOI:** 10.1155/2013/679308

**Published:** 2013-05-14

**Authors:** Archis R. Bhandarkar, Rohan Banerjee, Padmanabhan Seshaiyer

**Affiliations:** ^1^Thomas Jefferson High School for Science and Technology, Alexandria, VA 22312, USA; ^2^Mathematical Sciences, George Mason University, Fairfax, VA 22030, USA

## Abstract

Spontaneous pneumothorax, a prevalent medical challenge in most trauma cases, is a form of sudden lung collapse closely associated with risk factors such as lung cancer and emphysema. Our work seeks to explore and quantify the currently unknown pathological factors underlying lesion rupture in pneumothorax through biomechanical modeling. We hypothesized that lesion instability is closely associated with elastodynamic strain of the pleural membrane from pulsatile air flow and collagen-elastin dynamics. Based on the principles of continuum mechanics and fluid-structure interaction, our proposed model coupled isotropic tissue deformation with pressure from pulsatile air motion and the pleural fluid. Next, we derived mathematical instability criteria for our ordinary differential equation system and then translated these mathematical instabilities to physically relevant structural instabilities via the incorporation of a finite energy limiter. The introduction of novel biomechanical descriptions for collagen-elastin dynamics allowed us to demonstrate that changes in the protein structure can lead to a transition from stable to unstable domains in the material parameter space for a general lesion. This result allowed us to create a novel streamlined algorithm for detecting material instabilities in transient lung CT scan data via analyzing deformations in a local tissue boundary.

## 1. Introduction

Spontaneous pneumothorax is a form of sudden lung collapse closely associated with risk factors such as lung cancer and emphysema. The disorder represents a pressing clinical challenge, affecting between 20 and 40% of patients with major trauma [1]. The onset of pneumothorax has been attributed to the rupture of quasi spherical parenchymal lesions or air blebs of the visceral pleural membrane [2]. The rupture of these lesions in turn releases air into the pleural cavity, and the consequent pressure buildup leads to the collapse of the lung [3]. Because pneumothorax has a relatively large recurrence rate of approximately 54% in the first four years after surgery, wholly understanding and quantifying the mechanisms behind lesion rupture are important [4].

The etiology and trigger mechanisms behind lesion rupture, however, still remain elusive [2, 5]. We hypothesize that lesion rupture and instability are closely associated with elastodynamic strain of the visceral pleural membrane from pulsatile air flow and changes in the constitutive protein composition of tissue. In order to assess the validity of this hypothesis, we constructed a biomechanical model based on the principles of finite strain continuum mechanics and fluid-structure interaction. Our proposed model closely aligns with the behavior of true biological soft tissue through the coupling of membrane energy limitations and growth and remodeling driven by collagen-elastin dynamics. Through the use of an exhaustive ordinary differential equation stability analysis, we isolated several instability regions in the material parameter space of a general lesion.

**Figure 1:**
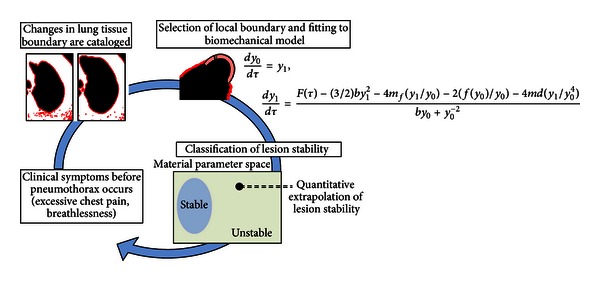
Outline of novel early diagnosis method. An outline of our novel diagnosis method. Based on the observation of certain symptoms, changes in the lung boundary are catalogued and material parameters are derived. The differential equation system is locally applied, and then a stability calculation is performed to determine the appropriate course of action.

Based on these mathematical results, we developed an algorithm to rapidly assay clinical lung CT scan data for structural instabilities. The algorithm is based on cataloging and processing local deformations in a tissue boundary isolated via Sobel Edge Detection. The local deformation of the boundary is then fit to our biomechanical model and registered as either stable or unstable based on whether or not the interpolated material parameters lie in the instability region.  Figure 1 shows a general outline of our novel early diagnostic method. In the future, the algorithm may be real time coupled with either ultrasound or CT scan data to quantify patient risk of pneumothorax earlier and improve the current diagnostic benchmark of only 75% lesion sensibility [6]. Unlike competitive diagnostic procedures for pneumothorax such as infrared thermography [1], our proposed method relies on detecting pathological hallmarks in tissue behavior before acute lung collapse actually occurs.

## 2. Methodology

Our study focused on the construction of a theoretically based biomechanical model for lung parenchymal lesion rupture and then the implementation of this model towards a streamlined algorithm for early pneumothorax diagnosis. We began with the theoretical development of a model for simple isotropic tissue deformation and then conducted several initial numerical investigations. Next, we derived mathematical instabilities for our system and then translated these mathematical instabilities to physically relevant structural instabilities via the incorporation of an energy limiter. We then incorporated collagen-elastin dynamics (dynamic alteration of the constitutive proteins in the tissue) to demonstrate how changes in tissue structure may lead to lesion rupture. Lastly, we developed an algorithm for determining lesion stability from CT scan data by fitting our biomechanical model onto tissue deformation data.

### 2.1. Mathematical Formulation

**Figure 2:**
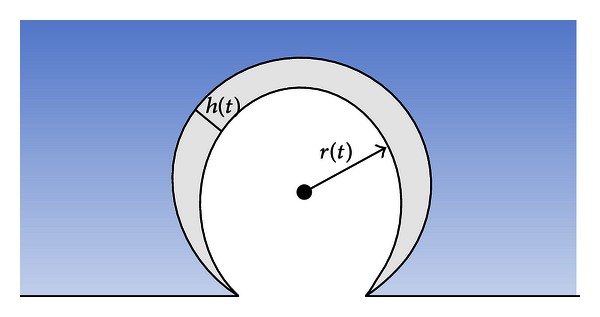
Schema of spherical parenchymal lesion. A schematic drawing of the spherical membrane geometry used as the basis for studying the growth of axisymmetric lung parenchymal lesions.

We modeled the parenchymal lesions with a spherical membrane geometry with thickness significantly smaller than the radius. Several parameters were needed in order to fully characterize the lesion geometry. The nondimensional stretch ratio *λ*(*t*) was defined as *r*(*t*)/*R*, where *r*(*t*) represents the deformed radius varying with time and *R* is the original undeformed radius. Assuming membrane incompressibility, the constant volume condition *V* = 4*πr* ^2^(*t*)*h*(*t*) implies that the deformed thickness is *h*(*t*) = *H*/*λ*(*t*)^2^, where *H* represents the original undeformed thickness.  Figure 2 depicts the geometry of these quasi-spherical lesions, including *r*(*t*) and *h*(*t*).

#### 2.1.1. Constitutive Parenchymal Wall Model

Based on the findings of Tai and Lee [7], who concluded that lung tissue exhibits less than 10% anisotropy of the mean deformation, we pursued an isotropic model for the lung parenchymal wall. We had based a collagen-only isotropic pseudostrain-energy function for the wall of the parenchymal lesion on the work of Denny and Schroter [8]. Previous works by Denny and Schroter [9, 10] had also considered collagen and elastin using separate models [9], which were enhanced with viscoelastic contributions [10]. We adapted the Denny-Schroter constitutive relationship for our 1D formulation through multiplication by the deformed thickness *h*:

(1) $$\begin{array}{c} w=\frac{H}{\lambda {\left(t\right)}^{2}}\left(c_{1}\log\left[1-\left(\frac{e^{E_{11}}-1}{c_{2}}\right)\right]+c_{3}E_{11}\right), \end{array}$$

where *c*_1_ and *c*_3_ are constants with dimensions N·m^−2^, *c*_2_ is dimensionless, and *E*_11_ = (*λ*(*t*)^2^ − 1)/2 (where **E** is the Green-Lagrangian strain tensor). Note that for simplicity, our model does not account for large stiffnesses for large strains. However, this is something that we hope to incorporate in future work. In order to facilitate the stability analysis and further calculations, this pseudostrain-energy function was approximated by a Taylor series centered about *E*_11_ = 0, yielding

(2) w=(−c1Hc2+c3H)E11+(−c1H2c22+3c1H2c2−2c3H)E112 +O[E113].

If the 2D deformation gradient **F** is diag(*λ*(*t*), *λ*(*t*)), then the Cauchy stress resultant tensor **T** for inner membrane stress can be represented by the following equation [11]:

(3) $$\begin{array}{c} T_{\alpha \beta }=\frac{1}{\det\;F}F_{\alpha \gamma }F_{\beta \delta }\frac{\partial w}{\partial E_{\gamma \delta }}+\frac{2\mu_{m}H}{\lambda {\left(t\right)}^{3}}\frac{d\lambda \left(t\right)}{dt},\;\alpha ,\beta ,\gamma ,\delta =1,2. \end{array}$$

Thus, the isotropic stress resultant *T* (*T*_11_) is given by the following:

(4) T=−c1Hc2+c3H+(−c1H2c22+3c1H2c2−2c3H)(λ(t)2−1) +2μmHλ(t)3dλ(t)dt.

#### 2.1.2. Model for Breathing Motion

We used a Fourier series developed by Jakuszkin [12] to model the pulsatile nature of breathing. The driven flow value of air through the lungs *Q* is

(5) $$\begin{array}{c} Q=\sum_{i=1}^{7}A_{i}\cos\left(2\pi f_{i}t+\phi_{i}\right). \end{array}$$

Table 1 contains the constants *A*_*i*_, *f*_*i*_, and *ϕ*_*i*_ [12].

Note that while we have employed a sinusoidal model for simplicity, we can extend the model to account for erratic breathing behavior, which is not considered here. The driven flow value is related to the pulsatile pressure through the relation Δ*P* = *R*_*b*_ *Q*, where *R*_*b*_ was the respiratory resistance of the bronchiole network. Because *R*_*b*_ is 1.0 mmHg·s·L^−1^ [13] and the original pressure was atmospheric pressure (760 mmHg), the final pressure series was as follows:

(6) $$\begin{array}{c} P_{\text{inner}}=760+\sum_{i=1}^{7}A_{i}\cos\left(2\pi f_{i}t+\phi_{i}\right). \end{array}$$

#### 2.1.3. Dynamic Equation System

We obtained the dynamic equation for the wall through linear momentum balance to yield

(7) $$\begin{array}{c} \rho_{m}hR\frac{d^{2}\lambda \left(t\right)}{dt^{2}}=P\left(t\right)-\frac{2T\left(\lambda \left(t\right)\right)}{\lambda \left(t\right)R}, \end{array}$$

where *ρ*_*m*_ is the density of the membrane and *P*(*t*) is the inner pressure minus the outer pressure (transmural pressure). The Navier-Stokes equations in spherical coordinates were used to model the pressure that the pleural fluid exerted on the lung parenchyma. The resultant pressure was as follows [14]:

(8) $$\begin{array}{c} P_{\text{outer}}=p_{\infty }+\rho_{f}R^{2}\left(\lambda \left(t\right)\frac{d^{2}\lambda \left(t\right)}{dt^{2}}+\frac{3}{2}{\left(\frac{d\lambda \left(t\right)}{dt}\right)}^{2}\right), \end{array}$$

where *p*_*∞*_ is a constant value representing the pressure at a significantly large distance from the lung and *ρ*_*f*_ is the density of the fluid. Note the absence of a viscous term as well as the same convention for the pressure at infinity as in the published work by Shah and Humphrey [14]. Combining the equations for the dynamic wall, the outer pressure, the dynamic radial stress which contributes the factor of *μ*_*f*_, and viscoelasticity, we arrived at the following differential equation for the air-tissue-pleural fluid system:

(9) (ρmHRλ(t)2+ρfR2λ(t))d2λ(t)dt2+32ρfR2(dλ(t)dt)2+4μfλ(t) ×dλ(t)dt+2T(λ(t))λ(t)R+4μmHλ(t)4Rdλ(t)dt=Pinner(t)−p∞,

where *μ*_*f*_ and *μ*_*m*_ are the dynamic viscosities of the pleural fluid and membrane, respectively.

#### 2.1.4. Nondimensionalization

Nondimensionalization is a process that helps to accommodate multiscale variations in the data. This technique is a common engineering practice which essentially simplifies multiphysics problems with different measured units involved. Because our final governing equations couple three different physical aspects (the air, the membrane, and the pleural fluid), this method was necessitated. Moreover, nondimensionalization recovers the characteristic properties of the stretch ratio and stretch rate. We followed this standard which is commonly used in published works on biomechanics such as Shah and Humphrey [14]. The following nondimensionalized quantities were required:

(10) $$\begin{array}{c} y_{0}\equiv \lambda \left(t\right),\;\;\tau \equiv t\sqrt{\frac{\left|c_{1}\right|H}{\rho_{m}R^{2}H}},\;\;b\equiv \frac{\rho_{f}R}{\rho_{m}H},\\ d\equiv \frac{H}{R},\;\;m_{f}\equiv \frac{\mu_{f}}{\sqrt{\rho_{m}\left|c_{1}\right|H^{2}}},\;\;m\equiv \frac{\mu_{m}}{\sqrt{\rho_{m}\left|c_{1}\right|H^{2}}},\\ F\left(\tau \right)\equiv \left(P_{\text{inner}}\left(t\right)-p_{\infty }\right)\frac{R}{c_{1}H},\;\;f\equiv \frac{T}{c_{1}H}. \end{array}$$

The resulting nondimensionalized equation system was as follows:

(11) dy0dτ=y1,dy1dτ=F(τ)−3by12/2−4mfy1/y0−2f(y0)/y0−4mdy1/y04by0+y0−2.

#### 2.1.5. Parameter Table

Table 2 is a complete collection of the relevant parameter values for the model.

### 2.2. Initial Numerical Investigations

**Figure 3:**
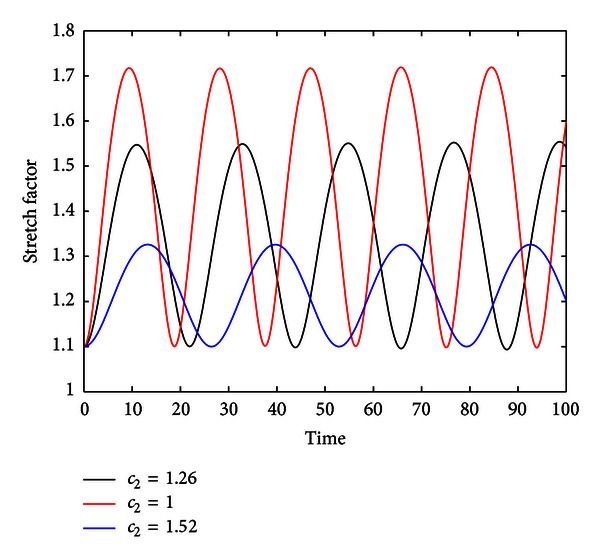
Stretch factor *λ*(*t*) for different stiffness constant (*c*_2_). This figure shows three plots of stretch factor with respect to dimensionless time for the stiffness constants *c*_2_ = 1.00,1.26, and  1.52. Variations in the stiffness constant clearly affect the amplitude and frequency of oscillations.

**Figure 4:**
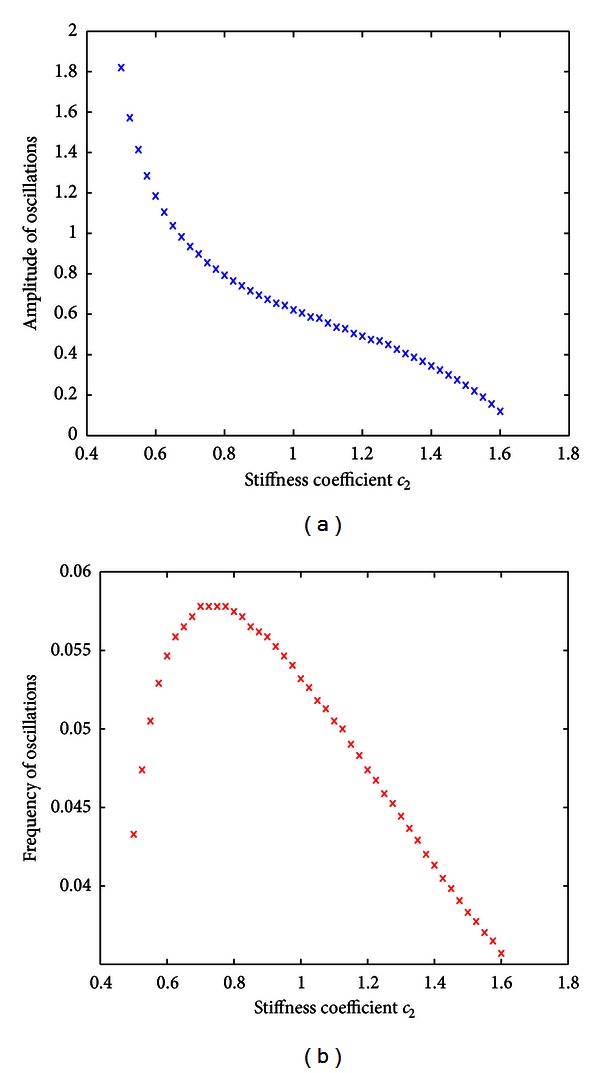
Quantified effects of stiffness constant (*c*_2_) on amplitude and frequency of oscillations. (a) The graph shows the effect of variations in stiffness constant on the amplitude of oscillations. The monotonically decreasing nature of the graph suggests that the stiffer the membrane, the smaller the amplitude. (b) The graph shows the effect of variations in stiffness constant on the frequency of oscillations with a maximum at *c*_2_ ≈ 0.78.

We conducted several numerical investigations in order to quantify both system dynamics and stability. The previous dynamical system was numerically evolved in MATLAB through a fourth-order explicit Runge-Kutta scheme. Plots of the stretch ratio *λ*(*t*) versus time reveal sustained stable oscillations of the membrane for normal lung parenchymal tissue parameters of the Denny-Schroter model: *c*_1_ = −22.5 × 10^5^, *c*_2_ = 1.26, and *c*_3_ = −7.8 × 10^5^. Variations in material stiffness proved an effective tool in analyzing the effects of parameter variation on system dynamics.  Figure 3 shows that as material stiffness increased in the model (for test values of *c*_2_ = 1.00,1.26, and  1.52), the amplitude of the oscillations decreased as one would expect intuitively.  Figure 4 shows that for the general range of *c*_2_ ∈ [.5,1.6], amplitude can be seen to monotonically decrease and begin to level off near zero. For the same window of stiffness constants, oscillation frequency reaches a peak near *c*_2_ ≈ 0.78 and then decreases monotonically from that value.

**Figure 5:**
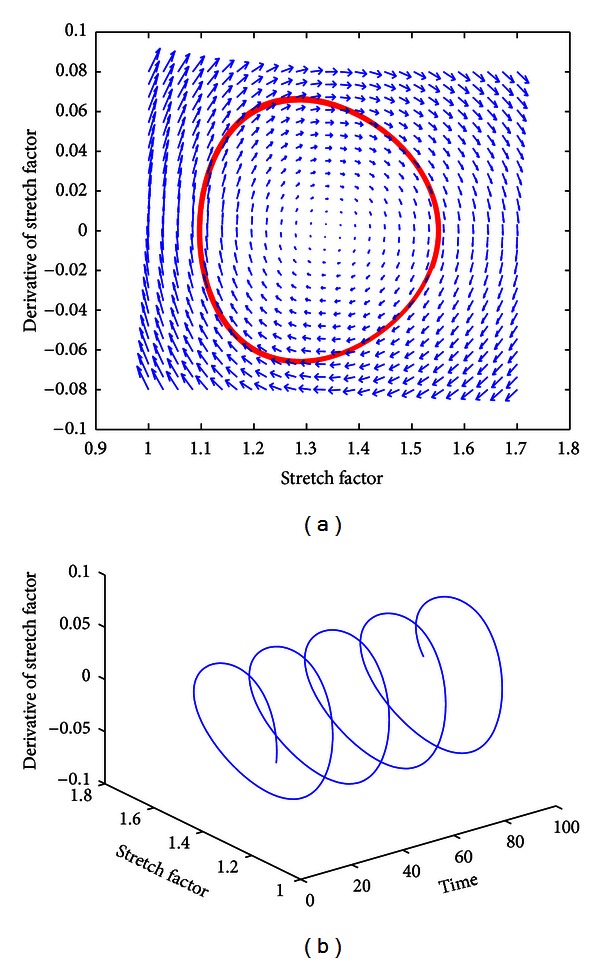
Numerically generated phase planes of the dynamical system. (a) The stable orbit and superimposed vector field demonstrated in the phase plane suggest the presence of an asymptotically stable equilibrium. (b) The 3D plot depicts the evolution of the stable orbit with respect to dimensionless time as further evidence of sustained stable oscillations. Refer to Section 2.4 for more information about physically unstable situations.

**Figure 6:**
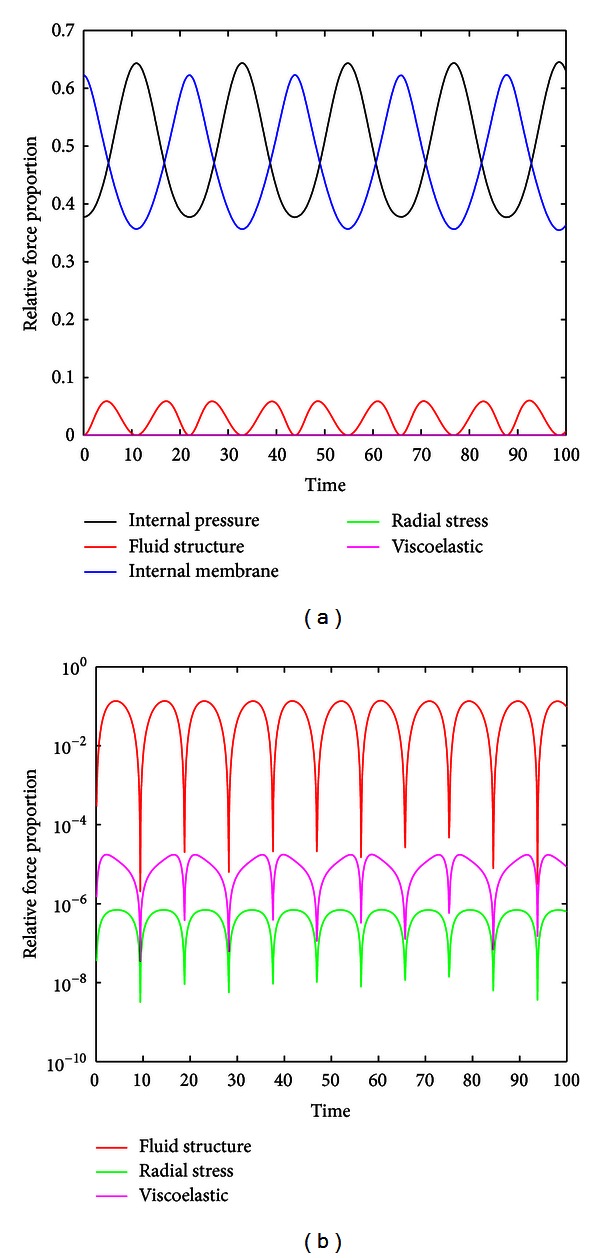
Relative force proportion graph. (a) The graph depicts the relative magnitude of the dimensionless forces at play over dimensionless time: pressure (*F*(*τ*)), radial stress (4*m*_*f*_ *y*_1_/*y*_0_), internal membrane (2*f*(*y*_0_)/*y*_0_), fluid structure (3*by*_1_^2^/2), and viscoelastic (4*mdy*_1_/*y*_0_). It is clear that internal pressure and internal membrane forces are the major driving forces behind the oscillations. (b) The three forces that contribute the least (radial stress, viscoelastic, and fluid structure) are plotted on semilogarithmic axes.

Figure 5 demonstrates that the system is stable even with minor perturbations in initial conditions for that class of material parameters. This suggests that there may be conditions where the bleb will not rupture.  Figure 6 is explained in the preceding paragraph as a relative force proportion graph, which illustrates the dynamic behavior of the different forces including internal pressure, fluid structure, internal membrane, radial stress, and viscoelasticity. The graph suggests that friction is not a significant factor leading to pneumothorax, and this aligns with other published works. The numerical phase plane analysis in Figure 5 of *λ*(*t*) versus $\dot{\lambda }\left(t\right)$ further suggests the existence of an orbit about a stable equilibria. The spiral nature of the superimposed vector field, representing the stretch rate $\dot{\lambda }\left(t\right)$ versus the derivative of the stretch rate $\ddot{\lambda }\left(t\right)$, suggests an asymptotically stable node at *λ*_eq_ ≈ 1.3 for normal lung parenchymal tissue parameters. A relative force proportion graph in Figure 6 reveals the forces of internal pressure and inner membrane stress as the major driving forces behind this oscillation, whereas the viscoelastic and fluid forces effects are negligible. Furthermore, several unstable regions had been isolated through variation of the material parameters such as for *c*_1_ = −22.5 × 10^5^, *c*_2_ = 1.26, and *c*_3_ = −1.39 × 10^6^, but a rigorous mathematical analysis had been called for to fully determine whether the instabilities were truly physical or just due to numerical effects.

### 2.3. Stability Results

#### 2.3.1. Determining System Equilibria

In order to simplify the following equilibria identification and stability analysis, we made the system autonomous by approximating *F*(*t*) ≈ *F* = *RP*/*c*_1_ *H*. The system is at an equilibrium for any point $\left(\lambda \left(t\right),\dot{\lambda }\left(t\right)\right)$ such that ${\dot{y}}_{0}=0$ and ${\dot{y}}_{1}=0$. This implies that $\dot{\lambda }\left(t\right)=0$ for any equilibrium point. The equilibrium stretch ratio *λ*_eq_ may be found by solving *F* − (*f*(*λ*(*t*))/*λ*(*t*)) = 0 as follows:

(12) $$\begin{array}{c} \lambda_{\text{eq}}=\frac{-\alpha \pm \sqrt{-4\beta \gamma +\alpha^{2}}}{2\gamma }, \end{array}$$

where *α* = *c*_1_ *c*_2_^2^ *F*, *β* = −*c*_1_ + 5*c*_1_ *c*_2_ − 6*c*_2_^2^ *c*_3_, and *γ* = *c*_1_ − 3*c*_1_ *c*_2_ + 4*c*_2_^2^ *c*_3_. For physically meaningful values of *λ*(*t*), the positive equilibrium point is the one of significance. For the normal lung parenchymal tissue parameters, *λ*_eq_^+^ = 1.3405 as suggested by the initial numerical investigation.

#### 2.3.2. Characterization of Unstable Nodes

We conducted a general ordinary differential equation (ODE) stability analysis on the physical system in order to evaluate a lung parenchymal lesion with arbitrary material parameters for physical instabilities. Given a system with Jacobian matrix *J*, ODE instability is prescribed for det⁡ *J* < 0  or  tr⁡ *J* > 0. For the previous dynamical system in *y*_0_ and *y*_1_, the Jacobian matrix about the positive equilibrium point reduces to

(13) J=[012Fγ(−4βγ+α2−α−4βγ+α2)α(−α+−4βγ+α2)2((4γ2/(−α+−4βγ+α2)2)+(b(−α+−4βγ+α2)/2γ))(2mf(α+−4βγ+α2)/β)−(64γ4dm/(−α+−4βγ+α2)4)((4γ2/(−α+−4βγ+α2)2)+(b(−α+−4βγ+α2)/2γ))].

The constraint of physically relevant material parameters imposes the inequalities *c*_1_ < 0,  *c*_2_ > 0, and *c*_3_ < 0, whereas the constraint of defined real elements of the Jacobian matrix imposes *β* ≠ 0,  *γ* ≠ 0, and *α* ^2^ ≥ 4*βγ*. Further note that from the definition of *F* = *AP*/*c*_1_ *H*, *α* is always greater than zero for physically relevant material constants (*A*, *H*, *P* > 0).

**Theorem 1**

For any values of *γ* and *β* such that *γ* < 0,  *β* > 0, and *γ* < *b* ^1/3^ *α* − *b* ^2/3^ *β*, the system is unstable because det⁡ *J* < 0.

The expression $\det\;J=-\partial {\dot{y}}_{1}/\partial y_{0}$ is less than zero for all *β*, *γ* such that $\mathrm{sgn}(\partial {\dot{y}}_{1}/\partial y_{0})>0$. The sign of this expression is in turn governed by the following three subexpressions. The subexpression of ${\left(-\alpha +\sqrt{-4\beta \gamma +\alpha^{2}}\right)}^{2}$ is consciously excluded from the sign analysis because it is invariantly positive.

**Lemma 2**

The sign of *β* and *γ* cannot both be positive for physically relevant parameters.

The lemma is proven by contradiction. By definition, *β* > 0 implies −*c*_1_ + 5*c*_1_ *c*_2_ − 6*c*_2_^2^ *c*_3_ > 0. Isolation of *c*_3_ reduces the inequality to *c*_3_ < *c*_1_(5*c*_2_ − 1)/6*c*_2_^2^. Likewise, *γ* = *c*_1_ − 3*c*_1_ *c*_2_ + 4*c*_2_^2^ *c*_3_ > 0 reduces to *c*_3_ > *c*_1_(3*c*_2_ − 1)/4*c*_2_^2^ and places bounds on *c*_3_. However, the inequality *c*_1_(3*c*_2_ − 1)/4*c*_2_^2^ < *c*_1_(5*c*_2_ − 1)/6*c*_2_^2^ reduces to 0 < *c*_1_(*c*_2_ + 1), which cannot be true from the restrictions of *c*_1_ < 0 and *c*_2_ > 0.

**Lemma 3**

The expression

(14) $$\begin{array}{c} -4\beta \gamma +\alpha^{2}-\alpha \sqrt{-4\beta \gamma +\alpha^{2}} \end{array}$$

is greater than zero for any value of *β*,  *γ* such that either *β* < 0 and *γ* > 0 or *β* > 0 and *γ* < 0. Similarly, the expression is less than zero for any value of *β*,  *γ* such that either *β* < 0 and *γ* < 0 or *β* > 0 and *γ* > 0.

The inequality $-4\beta \gamma +\alpha^{2}-\alpha \sqrt{\alpha^{2}-4\beta \gamma }>0$ reduces to $\alpha^{2}-4\beta \gamma >\alpha \sqrt{\alpha^{2}-4\beta \gamma }$. Because both sides of the inequality are positive, the inequality may be squared and the terms brought to one side to give (*α* ^2^ − 4*βγ*)(−4*βγ*) > 0. Because only values of *α*,  *β*,   and *γ* such that *α* ^2^ − 4*βγ* > 0 are considered, the expression is true *βγ* < 0. Thus, $-4\beta \gamma +\alpha^{2}-\alpha \sqrt{-4\beta \gamma +\alpha^{2}}>0$ for either *β* < 0 and *γ* > 0 or *β* > 0 and *γ* < 0. Likewise, $-4\beta \gamma +\alpha^{2}-\alpha \sqrt{-4\beta \gamma +\alpha^{2}}<0$ for *β* < 0 and *γ* < 0 or *β* > 0 and *γ* > 0.

**Lemma 4**

For physically relevant restriction of *b* > 0, the expression

(15) $$\begin{array}{c} \frac{4\gamma^{2}}{{\left(-\alpha +\sqrt{-4\beta \gamma +\alpha^{2}}\right)}^{2}}+\frac{b\left(-\alpha +\sqrt{-4\beta \gamma +\alpha^{2}}\right)}{2\gamma } \end{array}$$

is greater than zero for any value of *β*,  *γ* such that any one of the following three conditions are met: *γ* > 0 and *β* < 0; *γ* < 0 and *β* < 0; *γ* < *b* ^1/3^ *α* − *b* ^2/3^ *β*,  *γ* < 0, and *β* > 0.

For either the conditions of *γ* > 0 and *β* < 0 or *γ* < 0 and *β* < 0, the expression $b\left(-\alpha +\sqrt{-4\beta \gamma +\alpha^{2}}\right)/2\gamma$ is always greater than zero. Hence, because $4\gamma^{2}/{\left(-\alpha +\sqrt{-4\beta \gamma +\alpha^{2}}\right)}^{2}$ is invariantly positive, the entire expression (15) will be greater than zero for these conditions. Given *γ*,  *β* such that *γ* < 0 and *β* > 0, the inequality reduces to $8\gamma^{3}+b(-\alpha +\sqrt{-4\beta \gamma +\alpha^{2}})<0$ or $\gamma <-(b^{1/3}/2)\left(-\alpha +\sqrt{-4\beta \gamma +\alpha^{2}}\right)$. This in turn reduces to 4*γ*/*b* ^2/3^ − 4*α*/*b* ^1/3^ + 4*β* < 0 or *γ* < *b* ^1/3^ *α* − *b* ^2/3^ *β*.

**Lemma 5**

For physically relevant restriction of *b* > 0, the expression (15) is less than zero for any value of *β*,  *γ* such that *γ* < 0,  *β* > 0, and *γ* > *b* ^1/3^ *α* − *b* ^2/3^ *β*.

Either the conditions of *γ* > 0 and *β* < 0 or *γ* < 0 and *β* < 0 make the statement unconditionally false, leaving only the case of *γ* < 0,  *β* > 0. The fact that *γ* > *b* ^1/3^ *α* − *b* ^2/3^ *β* is proven likewise as in Lemma 4.

**Lemma 6**

The expression

(16) $$\begin{array}{c} \frac{2F\gamma }{\alpha } \end{array}$$

is greater than zero for all *γ* < 0 and less than zero for all *γ* > 0. By definition, *F* < 0 and *α* > 0, and thus the result follows.

The expression det⁡ *J* < 0 is true such that the product of expressions (14), (15), and (16) is positive. Upon coupling of the previous inequality systems, the only scenario that does not lead to contradictions is for (14), (15), and (16) all positive. This case implies that for *γ* < 0,  *β* > 0, and *γ* < *b* ^1/3^ *α* − *b* ^2/3^ *β* the system is unstable because det⁡ *J* < 0.

**Theorem 7**

For any values of *γ* and *β* such that *γ* < 0, *β* > 0, *γ* > *b* ^1/3^ *α* − *b* ^2/3^ *β*, and *β* > *αγ* ^2^(*dm*/*m*_*f*_)^1/3^ + *γ*(*dm*/*m*_*f*_)^2/3^, the system is unstable because tr⁡ *J* > 0.

The expression $\mathrm{tr}\;J=-\partial {\dot{y}}_{1}/\partial y_{1}$ is less than zero for all *β*, *γ* such that $\mathrm{sgn}(\partial {\dot{y}}_{1}/\partial y_{1})>0$. The sign of this expression is in turn governed by subexpressions (15) and (17).

**Lemma 8**

For physically relevant parameters (*m*_*f*_, *m*, *d* > 0),

(17) $$\begin{array}{c} \frac{2m_{f}\left(\alpha +\sqrt{-4\beta \gamma +\alpha^{2}}\right)}{\beta }-\frac{64\gamma^{4}dm}{{\left(-\alpha +\sqrt{-4\beta \gamma +\alpha^{2}}\right)}^{4}} \end{array}$$

is less than zero for all *β* < 0 or for *β* > 0,  *γ* < 0, and *β* > *αγ* ^2^(*dm*/*m*_*f*_)^1/3^ + *γ*(*dm*/*m*_*f*_)^2/3^.

For all *β* < 0, expression (17) is invariantly negative, and thus, the statements are unconditionally true. This leaves only the case of *β* > 0 and *γ* < 0. Manipulation of the expression yields

(18) $$\begin{array}{c} \frac{2m_{f}}{\beta }\left(\alpha +\sqrt{\alpha^{2}-4\beta \gamma }\right){\left(-\alpha +\sqrt{\alpha^{2}-4\beta \gamma }\right)}^{4}<64\gamma^{4}dm \end{array}$$

or ${\left(-\alpha +\sqrt{\alpha^{2}-4\beta \gamma }\right)}^{3}>-8\gamma^{3}dm/m_{f}$. This in turn yields *α* ^2^ − 4*βγ* > (*α*−2*γ*(*dm*/*m*_*f*_)^1/3^)^2^, which upon isolation of *β* gives the expression *β* > *αγ* ^2^(*dm*/*m*_*f*_)^1/3^ + *γ*(*dm*/*m*_*f*_)^2/3^.

**Figure 7:**
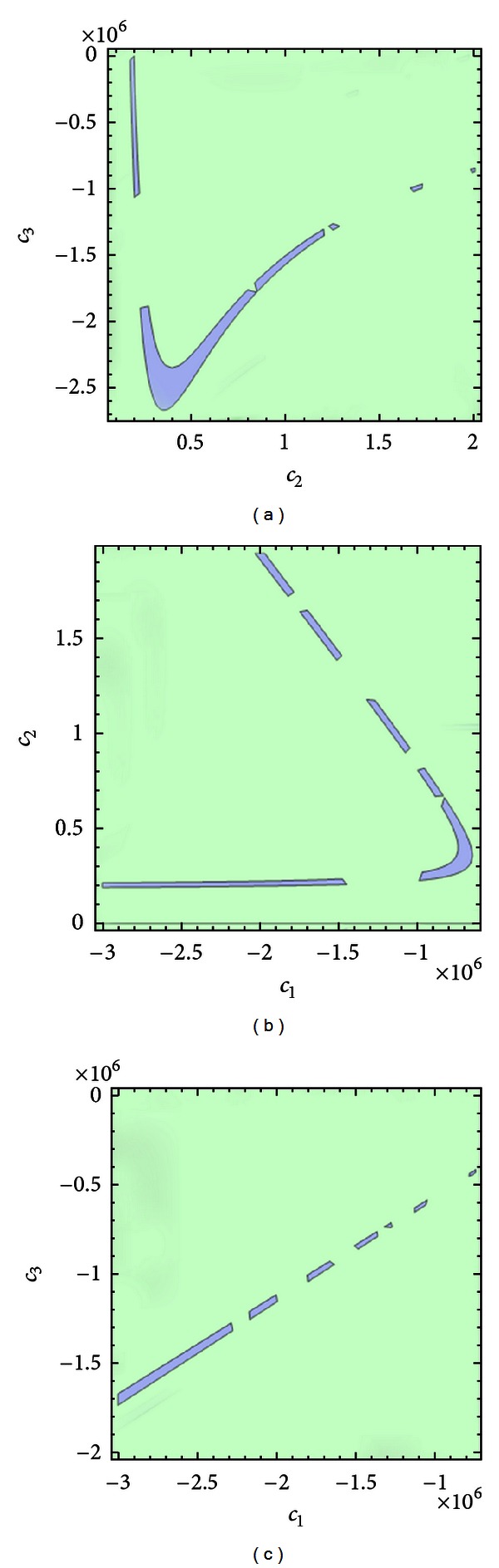
2D instability region portraits. The images show the regions of instability (blue) for each selection of two material parameters. The value of the third parameter in each graph is assumed to be the value from Denny and Schroter [8].

The expression tr⁡ *J* > 0 is true such that the product of expressions (15) and (17) is positive. For the signs of (15) and (17) to both be negative, Lemma 5 and Lemma 8 give *γ* < 0, *β* > 0, *γ* > *b* ^1/3^ *α* − *b* ^2/3^ *β*, and *β* > *αγ* ^2^(*dm*/*m*_*f*_)^1/3^ + *γ*(*dm*/*m*_*f*_)^2/3^. The case of expression (15) and (17) both greater than zero is consciously omitted from the proof because the analysis leads to an instability region already enclosed by Theorem 1.  Figure 7 shows 2D portraits of the regions obtained from these instability criteria for a certain selection of material parameters *c*_1_, *c*_2_, and *c*_3_.

### 2.4. Introducing Energy Limitations of Tissue

In order to accurately capture the biomechanics of lung parenchymal failure, we translated ODE instabilities to physical mechanical failure via the introduction of a finite energy limiter Φ(*Jm* ^−3^). Similar to Volokh and Vorp [19], we built a finite energy constitutive model Ψ based on the framework of the hyperelastic model *w* which was constructed as follows:

(19) $$\begin{array}{c} \Psi =H\Phi \left(1-\exp\left(\frac{-w}{H\Phi }\right)\right). \end{array}$$

Note that as *w* → *∞*, Ψ → *H*Φ a finite value of energy before rupture in contrast to the hyperelastic model where *w* may grow unbounded as *λ*(*t*) → *∞*. Similarly, in the limiting case *H*Φ ≪ *w*,  Ψ ≈ *w* as can be seen via Taylor expansion. Reformulating the Cauchy stress resultant tensor **T** given by [14] with the new strain-energy function yields

(20) $$\begin{array}{c} T_{\alpha \beta }^{\prime }=\frac{1}{\det\;F}F_{\alpha \gamma }F_{\beta \delta }\frac{\partial \Psi }{\partial E_{\gamma \delta }}+\frac{2\mu_{m}H}{\lambda {\left(t\right)}^{3}}\frac{d\lambda \left(t\right)}{dt},\;\alpha ,\beta ,\gamma ,\delta =1,2. \end{array}$$

Thus, the new isotropic stress resultant *T*′ (*T*_11_′) is given by the following:

(21) T′=(−c1Hc2+c3H+(−c1H2c22+3c1H2c2−2c3H)(λ(t)2−1)) ×exp⁡((c1H/c2−c3H)((λ(t)2−1)/2)HΦ+(c1H/2c22−3c1H/2c2+2c3H)((λ(t)2−1)2/4)HΦ) +2μmHλ(t)3dλ(t)dt.

**Figure 8:**
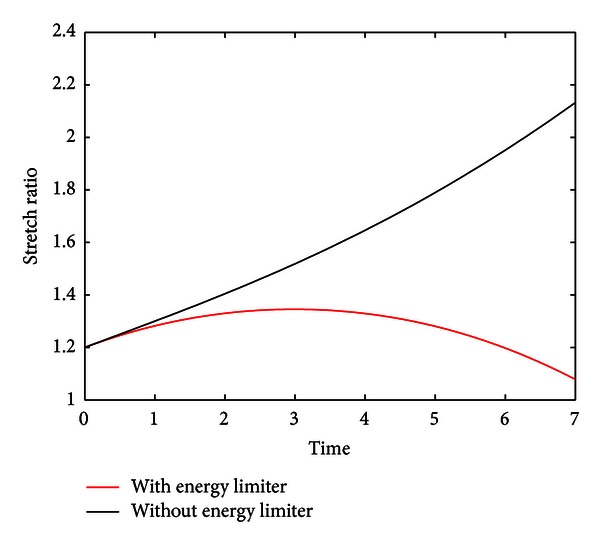
Effect of introduction of finite energy limiter. A point (*c*_1_, *c*_2_, *c*_3_) which satisfies the instability of requirement of Theorem 1  (−22.5 × 10^5^, 1.26, and − 1.39 × 10^6^) was used to test the effect of the finite energy limiter. As indicated by the red plot, the finite energy limiter curtails expansion to infinity and establishes a physical rupture point.

As demonstrated in Figure 8, numerical simulations with the new isotropic stress resultant demonstrate rupture midexpansion as opposed to expansion to infinity in the original constitutive model. 

### 2.5. Collagen-Elastin Dynamics

The final component of the proposed model for parenchymal lesion growth and rupture is a means by which stable mechanical configurations may enter unstable domains. The introduction of collagen-elastin dynamics (a dynamic protein framework of the tissue) elucidates mechanisms for how a stable lesion may eventually rupture. We introduced a new constitutive model through the creation of a linear combination of Denny and Schroter's [8] collagen-only strain energy function and Humphrey and Yin's [20] elastin-only strain energy function:

(22) w′=Ac(τ)(Hλ(t)2(c1log⁡[1−(eE11−1c2)]+c3E11))+Ae(τ)(bHλ(t)2(λ(t)−log⁡λ(t)−1)),w′≈(−Ac(τ)c1Hc2+Ac(τ)c3H)λ(t)2−12+(Ae(τ)bH2−Ac(τ)c1H2c22+3Ac(τ)c1H2c2−2Ac(τ)c3H)(λ(t)2−1)24,

where *A*_*c*_(*τ*) and *A*_*e*_(*τ*) are dimensionless quantities representing active collagen and elastin numbers. Similarly as before, we introduced a finite energy limiter Ψ′ to this constitutive model, and we recalculated the new isotropic strain tensor. Unlike the previous developments of the paper, collagen-elastin dynamics called for a return to the initial momentum balance due to the changing mass of the protein matrix. Given the mass of collagen *C* and elastin *E* defined by *ρ*_*c*_ *A*_*c*_ and *ρ*_*e*_ *A*_*e*_ (where *ρ*_*c*_ and *ρ*_*e*_ are equal to 5.10 kg·m^−3^ [21]), the new momentum balance was as follows:

(23) $$\begin{array}{c} \rho_{m}hR\frac{d^{2}\lambda \left(t\right)}{dt^{2}}+\frac{d\left(A_{e}\rho_{e}+A_{c}\rho_{c}\right)}{dt}v_{r}H=P\left(t\right)-\frac{2T\left(\lambda \left(t\right)\right)}{\lambda \left(t\right)R}, \end{array}$$

where *v*_*r*_(*t*) is the velocity at the boundary of an expanding biological membrane given by *Rdλ*(*t*)/*dt*. With the nondimensionalized term $m_{p}=(A/\sqrt{\rho_{m}|c_{1}|})(dC/dt+dE/dt)$, the first two differential equations of the dynamical system became

(24) dy0dτ=y1,dy1dτ=F(τ)−3by02/2−4mfy1/y0−2f(y0)/y0−4mdy1/y04−mpy1/y02by0+y0−2.

Based on histochemical evidence that had determined that collagen levels increase with increased stress and that elastin levels decrease because of elastolytic processes near rupture [22], we proposed the following differential equations for collagen-elastin dynamics:

(25) $$\begin{array}{c} \frac{dA_{c}}{dt}=\frac{k_{1}A_{c}}{{\left|\phi^{\prime }-\psi^{\prime }\right|}^{n}},\;\;\frac{dA_{e}}{dt}=\frac{-k_{2}A_{e}}{{\left|\phi^{\prime }-\psi^{\prime }\right|}^{n}}. \end{array}$$

**Figure 9:**
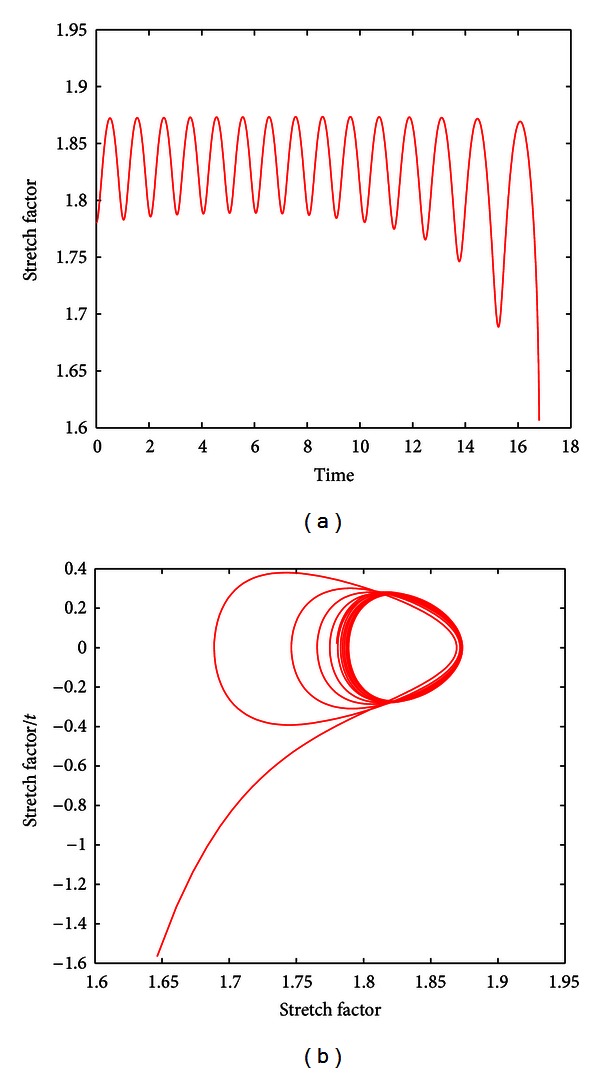
Collagen-elastin dynamics may lead to escapes from stable orbits. (a) The graph shows *λ*(*τ*) with the additional collagen-elastin dynamics calibrated with *A*_*c*_ = 100,  *A*_*e*_ = 50,  *k*_1_ = 1 × 10^7^,  *k*_2_ = 1 × 10^3^,  and *n* = 1. The point of rupture occurs at the final peak of oscillation at *τ* ≈ 16. (b) The phase plane reflects the transition from stability to instability as the trajectory veers off from stable orbits.

Because *k*_1_ and *k*_2_ are positive constants, we devised the differential equations such that little change occurs in the protein matrix in scenarios far from rupture (|*ϕ*′ − *ψ*′| ≫ 0) and larger changes occur as the membrane nears rupture. We numerically solved the series of four differential equations as a whole in MATLAB, revealing escapes from stable orbits for certain rate kinetics of the elastin-collagen dynamics that are depicted in Figure 9.

### 2.6. Parameter Estimation from *In Vivo* Data

In order to build a database for parameter estimation on the proposed model, we collected *in vivo* lung data in the form of CT scans from the VIA/I-ELCAP Public Lung Image Database, which were about 250 mm × 150 mm in size. The Sobel Edge Detection algorithm provided a means to quantify the deformation of the lung boundary and to extract material parameters from *in vivo* clinical data for the strain-energy function. This algorithm works by estimating the horizontal and vertical spatial gradients at the interior pixels, *G*_*x*_ and *G*_*y*_, through application of a matrix mask in order to detect edges. Pixels that meet a certain threshold for *G*  (|*G*_*x*_ | +|*G*_*y*_|) are counted as edges (Aybar, “Sobel Edge Detection”). This method was preferred over methods such as the Canny algorithm due to the coupling of speed and required accuracy for this problem. In order to facilitate the detection of the edges, we recolored the lung images to a 50% black and white scheme with 50% softening. After recoloration and softening, we ran the images through the Sobel algorithm implemented in Python, which returned a list of edge pixels for each image as coordinate pairs. We first transformed these pairs into a new coordinate system with the center of the image as the origin then categorized the pairs into subsections corresponding to sectors defined by angles. We then calculated values for the stretch factor, kinetic energy per square meter ((1/2)*ρ*_*m*_ *v* ^2^ *H*), and radial velocity in each radial direction.

**Figure 10:**
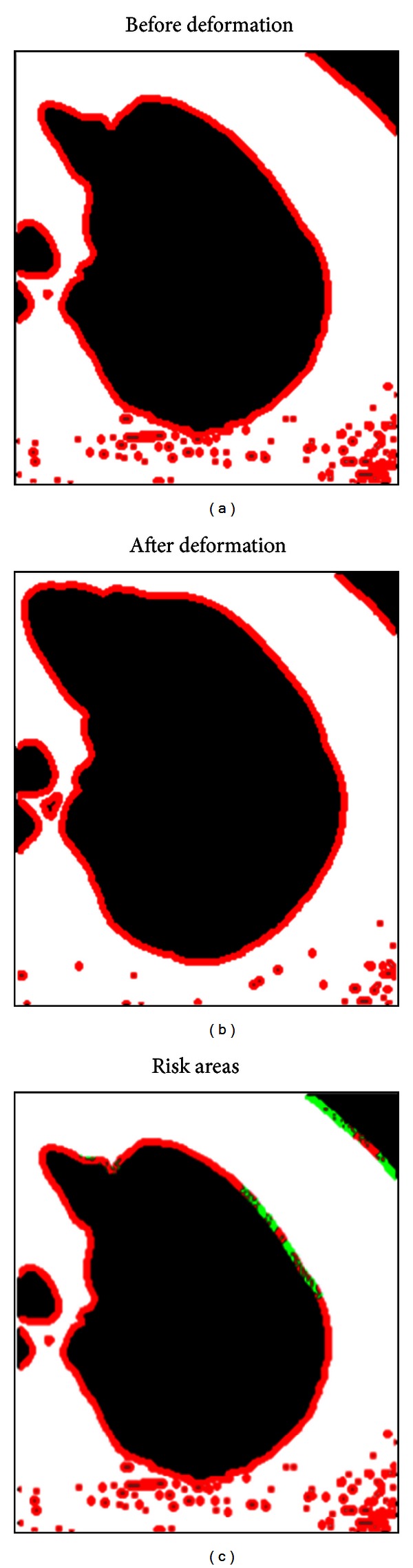
Edge detection and risk analysis. (a) and (b) show a lung before and after deformation, with red pixels indicating edge pixels detected by the Sobel Edge Detection algorithm. (c) shows the results of the nonlinear regression analysis performed on different sectors of the lung. Areas characterized as unstable, and thus at risk for lung collapse, are colored in red, whereas areas characterized as stable are colored in green.

Next, we applied nonlinear regression using the Levenberg-Marquadt algorithm on each subsection in order to extract the three material parameters *c*_1_, *c*_2_, and *c*_3_ as well as the corresponding coefficients of determination (*R* ^2^ values). We fit the velocity and kinetic energy data to the Denny-Schroter second-order Taylor series approximation. After we determined these parameters, they were classified as either stable or unstable corresponding to the previously derived stability criteria. Lastly, our program recolored the boundary of the lung parenchyma based on the stability of the corresponding subsections, with green corresponding to stable subsections and red corresponding to unstable subsections. The general schema for our computational algorithm is shown in Figure 10. Thus, our algorithm is streamlined towards interpolating material parameters from deformations in lung CT scan data and identifying specific areas of risk in the lung parenchyma.

Motion artifacts, while important and are often observed in CT data acquisition, are not considered in this paper. The images are represented using intensity values at various pixel locations. This paper used only one dataset as a proof of concept. Our algorithm looks at small sections of the lung boundary, allowing for the isotropic assumption to be valid. However, it must be noted that this assumption must be removed for more general studies. 

## 3. Conclusion and Future Directions 

Our proposed biomechanical model for lung parenchymal lesions demonstrates that elastodynamic strain of the visceral pleural membrane from pulsatile air flow and changes in the constitutive protein composition of tissue are key pathological factors in pneumothorax. Through proving general instability results, our study demonstrated that these factors biject to physically unstable domains in the material parameter space for a general lesion. Specifically, our discovery that certain rate kinetics of collagen-elastin dynamics may lead to unstable mechanical configurations aligns well with clinical studies, which have found that certain connective tissue diseases lead to spontaneous pneumothorax [23, 24]. Future work on the theoretical biomechanics aspect of our model will incorporate COMSOL multiphysics simulation software integrated with finite element mesh processing to solve the problems of nonaxisymmetry and anisotropic lesion growth. Also, it would be interesting to study how variations in the dynamic viscosities affect the behavior of parenchymal lesions. Clinical studies have indicated that gravity is a significant factor on the air accumulated in the pleural cavity. We hope to consider these effects in future work. Quantification of material properties will require efficient inverse parameter estimation studies, which we hope to incorporate in the future. Such studies will help us to develop efficient simulation tools [25, 26]. Other methods for lung deformation registration such as the dictionary learning approach used by Zhang et al. [27] will be considered. 

 Our algorithm for determining lesion stability from CT scan data relies on fitting our biomechanical model to deformations in a local tissue boundary. Using Sobel Edge Detection and CT scans from a lung image database, we could isolate unstable regions of local lung parenchymal tissue. The algorithm is a robust method for quantifying patient risk of pneumothorax and may be streamlined to work in the future with in vivo lung data collected from either ultrasound or CT scans. Overall, our computational solution for lung parenchymal lesion detection and patient-specific structural instability profiling is a feasible alternative diagnostic strategy and has the potential to surpass current diagnostic benchmarks for pneumothorax.

## Figures and Tables

**Table 1:** Lung Fourier series parameter table.

*i*	*A* (L·s^−1^)	*f* (Hz)	*ϕ*
1	1	0.156250	4.95
2	0.3	0.390625	3.82
3	0.25	0.859375	4.37
4	0.25	1.484375	3.67
5	0.175	2.421875	4.05
6	0.175	4.609375	4.13
7	0.125	8.046875	4.02

**Table 2:** List of parameters.

Parameter (variable)	Value	Citation
Material parameters (*c*_1_, *c*_2_, *c*_3_)	−22.5 × 10^5^ N·m^−2^, 1.26, −7.8 × 10^5^ N·m^−2^	Denny and Schroter, 2006 [8]
Undeformed lesion thickness (*H*)	10^−3^ m	Amjadi et al., 2007 [15]
Undeformed lesion radius (*R*)	10^−2^ m	Amjadi et al., 2007 [15]
Resistance of bronchiole network (*R*_*b*_)	1 mmHg·s·L^−1^	Ben-Tal, 2006 [13]
Pressure at infinity (*p*_∞_)	0 mmHg	Imposed
Pleural fluid viscosity (*μ*_*f*_)	1.39–1.57 × 10^−3^ Pa·s	Yetkin et al., 2007 [16]
Pleural fluid density (*ρ*_*f*_)	980.3 kg·m^−3^	Rubins and Manning, “Pleural Effusion Workup” [17]
Solid membrane viscosity (*μ*_*m*_)	7 × 10^−2^ Pa·s	Girnyk et al., 2006 [18]
Membrane density (*ρ*_*m*_)	1050 kg·m^−2^	Shah and Humphrey, 1999 [14]
Atmospheric pressure	760 mmHg	Ben-Tal, 2006 [13]
